# Factors associated with esophagojejunostomy stricture and its clinical impact after gastrectomy: a 10-year single-center study

**DOI:** 10.1007/s00423-026-04076-5

**Published:** 2026-05-25

**Authors:** Yonatan Lessing, Tal Inbar-Weissman, Orr Erlich-Feingold, Adi Litmanovitch, Fahim Kanani, Guy Lahat, Lior Orbach

**Affiliations:** 1 Division of Surgery, Tel Aviv Sourasky Medical Center (Ichilov), 6 Weizmann Street, Tel Aviv, 6423906 Israel; 2 Department of Transplantation, Beilinson Medical Center, Petach Tikva, 49100 Israel; 3 The Gray Faculty of Medicine and Health Sciences, Tel Aviv University, Tel-Aviv, 6997801 Israel

**Keywords:** Gastric Neoplasms, Gastrectomy, Anastomotic Stricture, Esophagojejunostomy, Postoperative Complications, Anastomosis

## Abstract

**Purpose:**

Esophagojejunostomy (EJ) stricture is a clinically significant complication following proximal and total gastrectomy, yet the underlying factors contributing to its development and its long-term implications remain poorly defined. This study evaluated the incidence, risk factors, and clinical impact of EJ stricture in patients undergoing gastrectomy for gastric adenocarcinoma.

**Methods:**

A retrospective cohort study was conducted that included all consecutive patients who underwent proximal or total gastrectomy with EJ reconstruction for gastric adenocarcinoma at a tertiary referral center (2014–2024). The primary outcome was stricture incidence, and the independent factors associated with stricture were identified. Secondary outcomes included management patterns, readmissions, complications, and survival. Logistic regression was used to identify factors independently associated with stricture; survival was evaluated using Kaplan-Meier analysis.

**Results:**

Among 139 patients, 15 (10.8%) developed EJ stricture; of these, three cases (20%) were later attributed to malignant recurrence. Baseline demographics, comorbidity profiles, operative approach, and tumor characteristics were similar between groups. Stricture patients were significantly more likely to have undergone circular anastomosis, either manual or stapled, compared with linear stapled EJ. Multivariable analysis identified circular EJ as the only factor independently associated with stricture (manual: OR 5.94, *p* = 0.013; stapled: OR 5.44, *p* = 0.018). Stricture patients had higher readmission rates at 30 days (53.3% vs. 17.7%, *p* = 0.004) and at 1 year (86.7% vs. 46.8%, *p* = 0.004). Median overall survival was significantly reduced among patients with stricture (18.2 vs. 29.3 months, *p* = 0.046). Malignant recurrence was identified in 20% of stricture cases.

**Conclusions:**

EJ stricture occurred in approximately 11% of gastrectomy patients and was strongly associated with circular anastomotic techniques. Stricture formation led to significantly increased readmissions and worse survival, partly due to malignant recurrence presenting as stricture. Linear stapled EJ may reduce the risk of stricture and should be considered when feasible.

## Introduction

Gastric cancer remains a major global health burden, ranking among the leading causes of cancer-related mortality worldwide despite improvements in diagnosis and treatment [1]. While distal gastrectomy has historically dominated surgical practice, the increasing incidence of proximal tumors and cancers involving the esophagogastric junction has expanded the use of proximal and total gastrectomy [2–4]. As survival improves, the priorities of surgical management have increasingly shifted toward balancing oncologic radicality with preservation of long-term nutritional function and postoperative quality of life [5–7].

Function-preserving procedures, particularly proximal gastrectomy with double-tract reconstruction (PG-DTR), have gained traction as multiple studies reporting favorable postoperative nutritional profiles compared with total gastrectomy, including improved hemoglobin recovery, reduced need for vitamin B12 supplementation, and better maintenance of skeletal muscle mass [8–10]. Randomized and propensity-matched studies have also shown similar short-term morbidity and long-term oncologic outcomes between PG-based reconstructions and total gastrectomy in appropriately selected patients [11–13].

Despite these advances, esophagojejunostomy (EJ) reconstruction remains a technically demanding step and a central determinant of postoperative morbidity after both proximal and total gastrectomy. Anastomotic stricture is particularly impactful: it may cause dysphagia, nutritional decline, recurrent hospitalizations, delays in adjuvant therapy, and diagnostic uncertainty when malignant recurrence is a concern. Prior meta-analyses in upper gastrointestinal surgery have demonstrated that anastomotic techniques, especially the choice between circular and linear stapling, substantially influence both stricture and leak rates [14–16]. Linear stapled EJ construction has consistently demonstrated lower stricture rates, potentially attributable to a wider anastomotic lumen and reduced localized tissue compression [15, 17].

Dedicated data describing risk factors for anastomotic stricture specifically after gastrectomy, however, remain limited. Moreover, benign strictures can mimic early locoregional recurrence in both symptoms and endoscopic appearance, complicating clinical decision-making and surveillance strategies. As minimally invasive and function-preserving gastrectomy techniques continue to expand, clarifying the incidence, predictors, and oncologic implications of postoperative EJ strictures has become increasingly important.

To address these gaps, we conducted a retrospective cohort study of all proximal and total gastrectomies performed at a high-volume tertiary center over ten years. The study aimed to (1) determine the incidence of anastomotic stricture after gastrectomy, (2) identify independent factors associated with stricture formation, with an emphasis on anastomotic technique, and (3) characterize management patterns and evaluate the impact of stricture on postoperative morbidity and survival.

## Materials and methods

### Study design and setting

We performed a retrospective cohort study at a single high-volume tertiary referral center. All consecutive adult patients who underwent proximal gastrectomy or total gastrectomy with EJ reconstruction for gastric adenocarcinoma between January 2014 and December 2024 were identified from institutional databases. The study was approved by the institutional review board of Tel Aviv Sourasky Medical Center (approval number: 0054-25-TLV), and informed consent was waived due to the retrospective design.

### Patient selection

Eligible patients were adults (≥ 18 years) with histologically confirmed gastric adenocarcinoma who underwent curative-intent proximal or total gastrectomy with esophagojejunostomy reconstruction. Patients were excluded if they had an R2 resection (macroscopically positive margins), underwent completion gastrectomy, or had non-adenocarcinoma pathology.

**Fig. 1:**
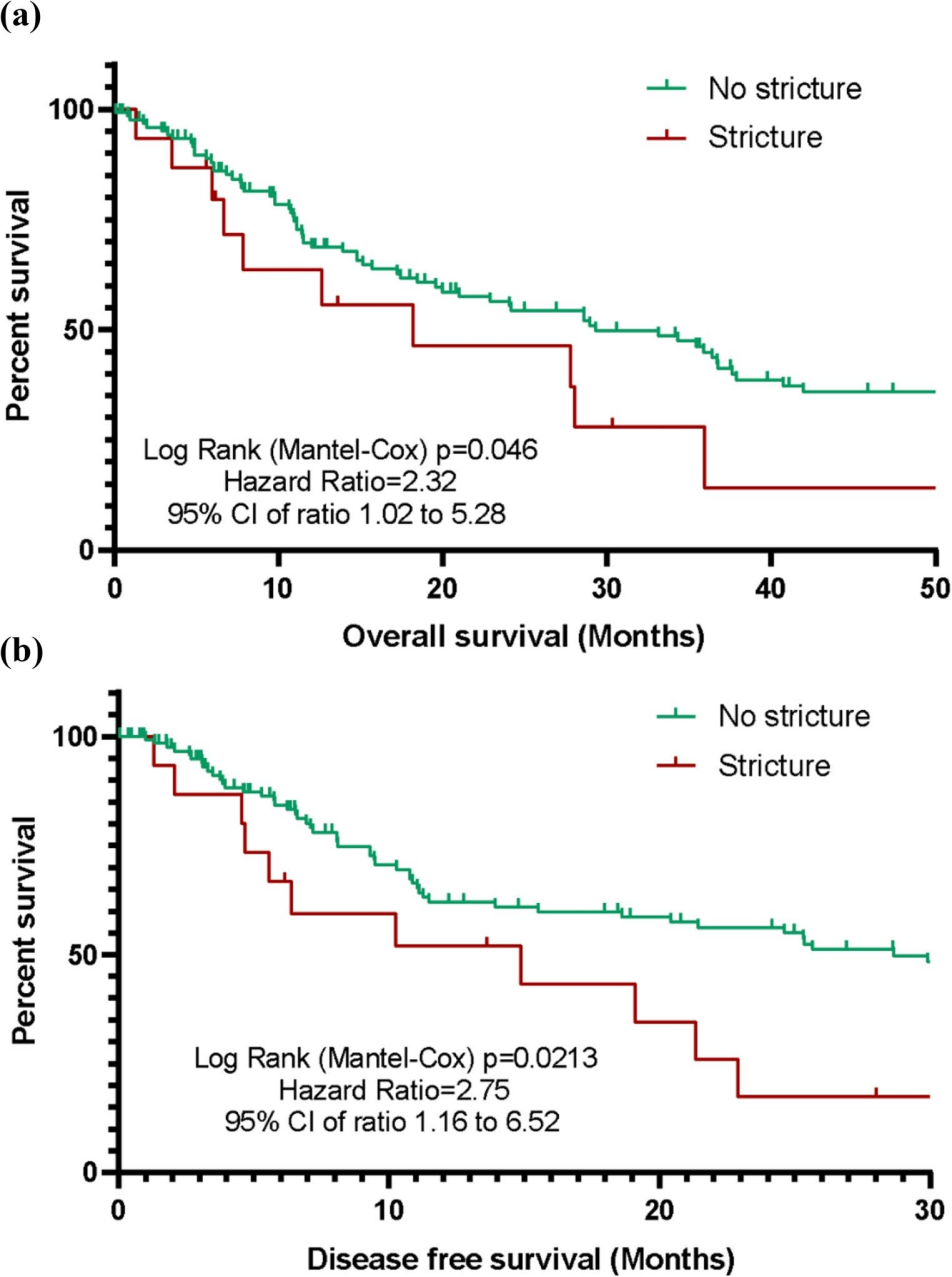
**a**: Kaplan-Meier overall survival curve by occurrence of stricture. **b**: Kaplan-Meier disease-free survival curve by occurrence of stricture

A total of 466 gastrectomies were performed during the study period. After applying the inclusion and exclusion criteria, 139 patients who underwent proximal or total gastrectomy with esophagojejunostomy reconstruction were included in the final analytic cohort (Fig. 1a). Palliative and urgent procedures were not excluded if esophagojejunostomy reconstruction was performed (Fig 1a).

### Definitions

#### Anastomotic stricture

Anastomotic stricture was defined as radiologic or endoscopic evidence of narrowing at the esophagojejunostomy in the presence of clinically relevant symptoms (e.g., dysphagia or intolerance of oral intake). Patients without both symptoms and confirmatory findings were not classified as having a stricture. In addition, patients presenting with symptoms suggestive of stricture but without objective evidence on endoscopic or radiologic evaluation were excluded from the stricture cohort.

#### Malignant stricture

Strictures were classified as malignant when endoscopic biopsy, cross-sectional imaging, or operative findings demonstrated local tumor recurrence at or near the anastomosis. These cases were retained within the overall cohort to reflect the full spectrum of clinically relevant postoperative anastomotic narrowing but were considered etiologically distinct from benign postoperative strictures.

#### Postoperative complications

Postoperative complications were graded according to the Clavien–Dindo classification. Anastomotic leak was defined based on clinical or radiologic evidence in accordance with standard criteria. Readmission was defined as any unplanned hospitalization within 30 days or within 1 year following the index operation.

#### Survival outcomes

Overall survival (OS) was defined as the interval from the date of surgery to death from any cause or last follow-up. Disease-free survival (DFS) was defined as the interval from the date of surgery to documented recurrence or death.

### Data collection

Data were extracted from electronic medical records, operative notes, endoscopy reports, and institutional cancer registries. Collected variables included patient factors (age, sex, body mass index, comorbidities, Charlson Comorbidity Index, preoperative serum albumin, neoadjuvant therapy); tumor characteristics (location, histologic type and grade, pathologic T and N stage, lymph node yield, margin status); operative variables (type of gastrectomy, surgical approach, urgency, reconstruction method, anastomotic technique, operative duration, intraoperative blood loss); postoperative outcomes (Clavien-Dindo complications, anastomotic leak, reoperation, intensive care unit admission, length of stay, and readmissions at 30 days and 1 year); stricture-specific data (time to diagnosis, diagnostic modality, need for dilation, number of dilation sessions, and additional interventions); and oncologic outcomes (recurrence pattern, malignant strictures, overall and disease-free survival). All variables correspond directly to the reported tables and figures of this manuscript.

Stricture-specific data were extracted from a dedicated institutional dataset, which prospectively captures all cases of postoperative esophagojejunostomy stricture, including time to diagnosis, diagnostic modality, management details, and the need for repeat interventions.

### Endpoints

The primary endpoint was the incidence of anastomotic stricture and the factors contributing to its development after proximal or total gastrectomy. Secondary endpoints included management patterns and the need for repeated intervention; the association between stricture and postoperative morbidity; 30-day and 1-year readmission rates; long-term overall and disease-free survival; and the incidence of malignant recurrence presenting as stricture.

### Statistical analysis

Continuous variables were summarized as mean (standard deviation) or median (interquartile range) and compared using the Student’s t-test or Mann-Whitney U test, as appropriate. Categorical variables were summarized as counts (percentages) and compared using χ² or Fisher’s exact test.

Variables with *p* < 0.10 on univariable analysis, along with clinically relevant factors, were entered into a multivariable binary logistic regression model to identify factors independently associated with anastomotic stricture. Survival outcomes were analyzed using the Kaplan-Meier method, and differences between groups were assessed with the log-rank test. Two-sided p-values < 0.05 were considered statistically significant. Statistical analyses were performed using IBM SPSS Statistics, version 29.0 (IBM Corp., Armonk, NY).

## Results

### Cohort characteristics

A total of 139 patients underwent proximal or total gastrectomy with EJ reconstruction during the study period. Fifteen patients (10.8%) developed an anastomotic stricture. Baseline demographic and preoperative characteristics were similar between patients with and without stricture. Age, BMI, comorbidity burden, and Charlson Comorbidity Index did not differ between groups.

Preoperative albumin was higher in the stricture group (41 vs. 38 g/L; *p* = 0.029), but this difference was not significant in multivariable analysis (Table 1).

**Table 1:** Demographics and pre-operative characteristics

	Total (*n*=139)	No stricture (*n*=124)	No stricture (*n*=15)	*p* (two-sided)
Female Gender– *n* (%)	52 (37.4)	43 (34.7)	9 (60.0)	.056
Age - Median (IQR)	68.3 (59.9–76.7)	68.4 (59.4–77.4)	68.0 (63.4–76.0)	.873
BMI - Median (IQR)	24.9 (22.5–27.7)	24.9 (22.5–28.0)	24.5 (21.6–27.3)	.717
Co-morbidities: *n* (%)				
HTN	67 (48.2)	57 (46.0)	10 (66.7)	.130
DM	29 (20.9)	27 (21.8)	2 (13.3)	.737^⸸^
Ischemic heart disease	27 (19.4)	24 (19.4)	3 (20.0)	1.000^⸸^
COPD	8 (5.8)	7 (5.6)	1 (6.7)	1.000^⸸^
Charlson comorbidity index score - Median (IQR)	1.0 (0.0–2.0)	1.0 (0.0–2.0)	1.0 (0.0–2.0)	.540
Preop loss of weight – *n* (%)	81 (58.3)	69 (55.6)	12 (80.0)	.071
Preop chemotherapy - *n* (%)	56 (40.3)	47 (37.9)	9 (60.0)	.099
Preop Albumin - Median (IQR)	39 (35–42)	38 (35–41)	41 (38–44)	**.029**
Preop Hemoglobin - Median (IQR)	11.3 (10.2–12.5)	11.3 (10.4–12.5)	10.9 (9.9–12.3)	.309

*IQR* Inter Quartile Range; *BMI* Body Mass Index; *HTN* Hypertension; *DM* Diabetes Mellitus; *COPD* Chronic Obstructive Lung Disease; *Preop* Pre-operative

^⸸^Fisher exact test

### Operative and pathological characteristics

Operative and pathological characteristics are summarized in Table 2. The majority of procedures were performed via an open approach (78.4%), with a similar distribution between groups. There were no differences in operative duration, intraoperative blood loss, or urgency of surgery. The proportions of proximal and total gastrectomy were comparable between patients with and without stricture.

**Table 2:** Surgical and pathological characteristics

	Total (*n*=139)	No stricture (*n*=124)	Stricture (*n*=15)	*p* (two-sided)
Urgent surgery – *n* (%)	4 (2.9)	3 (2.4)	1 (6.7)	.370^⸸^
Palliative surgery	12 (8.6)	11 (8.9)	1 (6.7)	1.000^⸸^
Surgical approach – *n* (%)				
Open surgery	109 (78.4)	99 (79.8)	10 (66.7)	.260
Minimally invasive Surgery	30 (21.6)	25 (20.2)	5 (33.3)	
Type of anastomosis – *n* (%)				
Manual anastomosis	15 (10.8)	11 (8.9)	4 (26.7)	.013
Stapled circular anastomosis	18 (12.9)	13 (10.5)	5 (27.8)	
Stapled linear anastomosis	82 (59.0)	77 (62.1)	5 (6.1)	
Type of lymph node dissection – *n* (%)				
D1	49 (35.3)	44 (35.5)	5 (33.3)	.777
D2	74 (53.2)	65 (52.4)	9 (60.0)	
Not available	16 (11.5)	15 (12.1)	1 (6.7)	
Pathological staging *n* (%)				
T1–2	39 (34.2)	36 (36.4)	3 (20.0)	.36
T3	42 (36.8)	36 (36.4)	6 (40.0)	
T4	33 (28.9)	27 (27.3)	6 (40.0)	
pN stage				
N0	44 (38.6)	39 (39.4)	5 (33.3)	.87
N1–2	31 (27.2)	27 (27.3)	4 (26.7)	
N3	39 (33.6)	33 (33.3)	6 (40.0)	
Pathological stage group				
I–II	28 (24.6)	24 (24.2)	4 (26.7)	.78
III	84 (73.7)	73 (73.7)	11 (73.3)	
IV	4 (3.5)		4 (4.0)	
No. of Lymph nodes resected – Median (IQR)	24 (14–32)	22 (14–32)	27 (17–37)	.231
Metastatic lymph node involvement - *n* (%)	88 (65.2)	78 (64.5)	10 (71.4)	.770⸸

*SD* Standard Deviation; *IQR* Interquartile Range

^⸸^Fisher exact test

Pathologic tumor characteristics were also similar between groups. There were no significant differences in pT stage (*p* = 0.36), nodal status (*p* = 0.87), or overall pathological stage group (*p* = 0.78).

### Anastomotic technique

Anastomotic technique differed between groups. Patients who developed strictures more frequently underwent circular-stapled or hand-sewn anastomoses, whereas linear-stapled esophagojejunostomy was more commonly used in patients without stricture. Other operative variables, including the extent of lymphadenectomy and reconstruction configuration, were not associated with stricture formation.

### Stricture presentation and management

Stricture-specific data were extracted from a dedicated institutional dataset, which prospectively captures all cases of postoperative esophagojejunostomy stricture identified during routine clinical follow-up. The dataset includes time to diagnosis, diagnostic modality, management details, and the need for repeat interventions, and is maintained using standardized data collection from endoscopy reports, imaging studies, and clinical records.

Strictures were diagnosed primarily by upper endoscopy (73.3%), with the remainder identified on contrast studies or cross-sectional imaging. The median time from surgery to stricture diagnosis was 104.5 days (IQR 70–155).

Endoscopic balloon dilation was performed in 9 of 15 patients (60%), and clinical improvement was achieved in 8 of 9 (88.9%). The median number of dilation sessions was 3 (IQR 1.75–4).

Three strictures (20%) were attributed to malignant recurrence at or near the anastomosis and were managed with endoscopic stent placement. Notably, all patients in the stricture group had undergone R0 resection at the index operation, and none had received radiotherapy prior to stricture diagnosis, making treatment-related fibrosis unlikely.

Of the remaining three patients (20%) with benign strictures who did not undergo dilation, one underwent eventual revisional surgery due to suspected anastomotic torsion, one had a severe non-traversable inflammatory stricture that precluded dilation and was managed with enteral feeding access, and one underwent attempted dilations without clinical improvement and was subsequently lost to follow-up. There was no significant difference in time to diagnosis between malignant and benign strictures (192 [IQR 40–311] vs. 104.5 [IQR 70–155] days, *p* = 0.65).

### Postoperative morbidity and readmissions

Postoperative outcomes are presented in Table 3. Rates of major complications (Clavien–Dindo grade ≥ III), anastomotic leak, reoperation, bleeding, respiratory complications, and surgical site infection were similar between patients with and without stricture.

**Table 3:** Postoperative course and outcomes

	Total (*n*=466)	No stricture	Stricture	*p* (two-sided)
Post-operative complications - *n* (%)				
Clavien-dindo ≥ 3	26 (18.7)	23 (18.5)	3 (20.0)	1.000^⸸^
Re-operation	13 (9.4)	12 (9.7)	1 (6.7)	1.000^⸸^
Surgical Site Infection	13 (9.4)	13 (10.5)	0 (0.0)	.360^⸸^
DGE / enteral feeding intolerance	8 (5.8)	7 (5.6)	1 (6.7)	1.000^⸸^
Anastomotic leak / intra-abdominal infection / GI fistula	39 (28.1)	36 (29.0)	3 (20.0)	.557^⸸^
Bleeding	5(3.6)	5 (4.0)	0 (0.0)	1.000^⸸^
Respiratory complication	27 (19.4)	26 (21.0)	1 (6.7)	.302^⸸^
Other non-surgical complications	39 (28.1)	34 (27.4)	5 (33.3)	.761^⸸^
ICU admission - *n* (%)	54 (38.8)	52 (41.9)	2 (13.3)	.032
Length of stay, days (Mean ± SD)	13 (10–22)	13 (10–22)	13 (9–20)	.488
30-day mortality - *n* (%)	3 (2.2)	3 (2.4)	0 (0.0)	1.000^⸸^
60-day mortality - *n* (%)	6 (4.3)	5 (4.1)	1 (6.7)	.505^⸸^
90-day mortality - *n* (%)	7 (5.1)	6 (4.9)	1 (6.7)	.562^⸸^
Returning to chemotherapy - *n* (%)	61 (43.9)	52 (41.9)	9 (60.0)	.183
30 days Re-admission	30 (21.6)	22 (17.7)	8 (53.3)	.004^⸸^
1 year Re-admission	71 (51.1)	58 (46.8)	13 (86.7)	.004
Median overall survival – Median (IQR)	28.6 (10.9–95.4)	29.3(10.9–108.5)	18.2 (6.7–36.0)	.046^⸹^

*DGE* Delayed Gastric Emptying; *GI* Gastrointestinal; *ICU* Intensive Care Unit; *SD* Standard Deviation; *IQR* Interquartile Range

^⸸^Fisher exact test, ^⸹^Log rank test

Patients with stricture had higher 30-day (53.3% vs. 17.7%; *p* = 0.004) and 1-year (86.7% vs. 46.8%; *p* = 0.004) readmission rates. ICU admission was less frequent in the stricture group (*p* = 0.032).

The median time to stricture diagnosis was 104.5 days (IQR 70–155). Causes of 30-day readmissions in patients with stricture were heterogeneous and included upper gastrointestinal symptoms (e.g., dysphagia or intolerance of oral intake), infectious complications, metabolic disturbances, and unrelated medical events.

### Survival

Kaplan–Meier survival curves are shown in Fig. 1a and 1b. Median overall survival was shorter in patients who developed a stricture than in those who did not (18.2 vs. 29.3 months; *p* = 0.046). Disease-free survival showed a similar trend toward worse outcomes in the stricture group, paralleling the higher proportion of malignant recurrence presenting as stricturing disease.

All patients who developed stricture in our cohort were subsequently diagnosed with disease recurrence. Among those who did not initially present with a malignant stricture, recurrence patterns included isolated lymphadenopathy in one patient, liver metastases in two patients, and lung metastases in two patients, while the remaining patients developed ascites with radiologic features consistent with peritoneal dissemination. These findings indicate heterogeneous recurrence patterns without a single dominant pathway.

### Multivariable analysis

Results of the multivariable logistic regression are shown in Table 4. The type of anastomosis was the only factor independently associated with stricture. Compared with linear stapled EJ, both hand-sewn anastomosis (odds ratio [OR] 5.94; 95% confidence interval [CI] 1.45–24.34; *p* = 0.013) and circular stapled anastomosis (OR 5.44; 95% CI 1.34–22.08; *p* = 0.018) were associated with increased odds of stricture. No demographic, nutritional, surgical approach-related, or oncologic variables were independently associated with stricture formation.

**Table 4:** Binary logistic regression analysis for the occurrence of stricture

Factor	Sig.	HR	95.0% CI for HR	
			Lower	Upper
Hand-sewn anastomosis	.013	5.94	1.45	24.34
Stapled circular anastomosis	.018	5.44	1.34	22.08

## Discussion

This study assessed the incidence, risk factors, and clinical impact of esophagojejunostomy stricture following proximal or total gastrectomy for gastric adenocarcinoma. Among 139 patients, 10.8% developed postoperative stricture, and the principal finding was that anastomotic technique, specifically the use of circular stapled or manually constructed EJ anastomoses, was the only factor independently associated with stricture. No demographic, nutritional, operative, or tumor-related characteristics were independently associated with stricture risk. These findings highlight the critical role of anastomotic geometry and mechanics in maintaining postoperative anastomotic patency.

### Comparison with existing literature

Our observed stricture rate is consistent with previous reports, which typically range from 5% to 15% depending on the extent of resection, reconstruction method, and surveillance intensity [14, 15, 18]. Large meta-analyses in upper gastrointestinal surgery have demonstrated that linear-stapled EJ anastomoses are associated with significantly lower stricture rates than circular-stapled anastomoses [14–16]. Several mechanisms have been proposed, including a wider anastomotic diameter, more uniform tissue compression, and reduced ischemic injury at the EJ staple line [17, 19]. These technical considerations align closely with the findings of our study, in which circular anastomoses-both manually constructed and stapled-carried a five- to six-fold increased odds of stricture compared with linear stapling.

Although our investigation was not intended as a comparison between proximal and total gastrectomy, our findings are broadly consistent with recent work evaluating reconstruction safety in these settings. The KLASS-05 randomized trial, which compared PG-DTR with laparoscopic total gastrectomy, reported no differences in short-term morbidity, anastomotic complications, or early postoperative symptom scores despite the structural complexity of PG-DTR [11]. Importantly, neither KLASS-05 nor the present study demonstrated higher rates of EJ-related complications when an additional GJ anastomosis was required. This suggests that, when performed by experienced teams, the technical safety of EJ reconstruction is not primarily dependent on the extent of gastric resection but rather on the specific method used to construct the anastomosis.

Anastomotic outcomes appear to be influenced by surgeon experience and institutional volume. Higher rates of leak and stricture have been reported during the early learning curve of laparoscopic total gastrectomy, potentially related to technical challenges in achieving a tension-free esophagojejunostomy within a constrained operative field [20, 21].

In contrast, centers performing high annual volumes of minimally invasive gastric cancer surgery report EJ leak rates of 1% to 3% and stricture rates in the single digits [7, 10, 18]. In this cohort, the majority of procedures were performed via an open approach, reflecting practice patterns during the study period. Although minimally invasive gastrectomy is increasingly adopted, particularly for total gastrectomy, its uptake has historically been gradual. In our analysis, the surgical approach was not associated with stricture formation.

### Clinical significance of stricture formation

The clinical impact of anastomotic stricture in our cohort was substantial. Patients who developed strictures had higher 30-day and 1-year readmission rates, consistent with the resource-intensive nature of recurrent dysphagia, dehydration, and nutritional compromise. These findings echo reports from other centers demonstrating that postoperative stricture frequently necessitates repeated endoscopic dilation, supplemental nutritional support, and prolonged postoperative surveillance [18, 22–24].

Overall survival was significantly worse among patients with stricture. While stricture itself is unlikely to directly influence long-term survival, three cases (20%) were attributable to malignant recurrence at or near the anastomosis. Although this proportion is slightly higher than previously reported estimates, the small number of events limits interpretation.

Importantly, all index resections were R0, and none of these patients had received radiotherapy prior to stricture diagnosis, making residual disease and treatment-related fibrosis unlikely explanations. These cases therefore likely represent a distinct clinical entity from benign anastomotic strictures related to technical or healing factors.

Given the limited number of events, we elected to retain malignant strictures in the analysis to reflect the full spectrum of clinically relevant postoperative anastomotic narrowing encountered in practice, while explicitly distinguishing them from benign strictures. The absence of a significant difference in time to diagnosis between malignant and benign strictures further supports that their inclusion is unlikely to have materially influenced the identification of factors associated with stricture formation.

Further insight is provided by recurrence patterns in patients who developed stricture. In our cohort, all patients with stricture ultimately experienced disease recurrence. Among those who did not initially present with a malignant stricture, recurrence manifested as lymph node disease in one patient, liver metastases in two patients, and lung metastases in two patients, while the remaining patients developed ascites with radiologic features consistent with peritoneal dissemination. These findings suggest that, in some cases, stricture may be associated with underlying disease recurrence rather than representing a purely technical complication. This heterogeneity suggests that the association between stricture and reduced survival is likely mediated by underlying oncologic progression rather than a specific recurrence phenotype.

At the same time, the variability in recurrence patterns indicates that no single pathway predominates, reinforcing the need for comprehensive oncologic evaluation in patients presenting with postoperative dysphagia.

### Role of preoperative staging and lymph node assessment

Differences in pathological nodal stage between stricture and non-stricture patients in our cohort most likely reflect underlying tumor biology rather than any causal association with stricture formation. This interpretation is consistent with well-established evidence that preoperative clinical staging frequently underestimates true nodal disease in gastric cancer. Berlth et al. demonstrated substantial limitations in the accuracy of CT, EUS, and PET for N-staging, with routine understaging in clinical practice [25]. Similarly, Kim et al. reported that clinical nodal assessment underestimated pathological N-category in a significant proportion of patients, particularly those with more advanced tumors [26].

Such discrepancies have practical consequences for surgical planning, as unsuspected involvement of lymph node stations 5 or 6 may necessitate total gastrectomy rather than proximal resection [27]. Emerging techniques, including sentinel node navigation and ICG-guided lymphatic mapping, offer promise for improving nodal assessment, although no standardized global approach currently exists [28–30].

### Factors associated with stricture formation

Beyond anastomotic technique, several variables are commonly proposed to contribute to stricture formation, including neoadjuvant therapy, ischemia at the EJ site, excessive tension, and postoperative complications such as leaks or abscesses [17, 21, 31]. In our study, none of these factors independently predicted stricture. Importantly, we did not observe an association between neoadjuvant therapy and stricture formation, consistent with recent literature suggesting that neoadjuvant chemotherapy does not significantly impair anastomotic healing in gastric cancer surgery [31, 32].

Preoperative albumin was slightly higher among patients who developed strictures, though this association did not persist in multivariable analysis and likely reflects random variation due to small sample size. Previous studies have suggested that hypoalbuminemia correlates with anastomotic complications; however, these findings are inconsistent across the literature and may not apply uniformly to EJ-specific stricture [33].

### Strengths and limitations

This study has several strengths, including a decade-long cohort from a high-volume tertiary cancer center, standardized definitions for stricture, and operative documentation that allowed classification of anastomotic technique in the majority of cases.

However, several limitations should be acknowledged. First, the retrospective design introduces potential selection bias and unmeasured confounding; therefore, observed associations should not be interpreted as causal.

Second, the number of stricture events was relatively small (*n* = 15), limiting statistical power and resulting in wide confidence intervals, particularly in multivariable analysis.

Third, although operative documentation was available, complete technical details were not uniformly captured. The anastomotic technique could not be definitively classified in approximately 17% of cases, and granular variables such as circular stapler diameter were not consistently recorded, potentially introducing misclassification bias and limiting more detailed technical analysis.

Fourth, the predominance of open surgical approaches (78%) may limit the generalizability of our findings to centers where minimally invasive gastrectomy is more commonly performed.

Finally, variation in surgical practice - including the extent of lymphadenectomy (with a substantial proportion of D1 dissections) and the use of neoadjuvant therapy - may differ from contemporary guideline-based standards. These patterns likely reflect evolving practice over the study period, as well as heterogeneity in clinical staging and multidisciplinary decision-making. The absence of structured clinical staging (cTNM) further limits assessment of the appropriateness of treatment strategies relative to disease.

Although malignant strictures were identified and explicitly distinguished from benign strictures, the small number of malignant cases (*n* = 3) limits conclusions regarding their clinical behavior and impact on outcomes. These limitations should be considered when interpreting the magnitude of observed associations.

## Conclusions

In this single-center cohort, anastomotic stricture occurred in approximately 11% of patients following proximal or total gastrectomy. Among all clinical, operative, and oncologic variables examined, anastomotic technique was the only factor independently associated with stricture formation, with non-linear approaches (circular stapled and hand-sewn esophagojejunostomy) associated with increased odds of stricture. Strictures were associated with increased readmissions and worse overall survival, partly due to a substantial proportion representing malignant recurrence. These findings support the preferential use of linear stapled EJ techniques when feasible and underscore the importance of vigilant, biopsy-directed evaluation of postoperative dysphagia. Further prospective studies are warranted to refine risk stratification and to establish standardized algorithms for diagnosis and management of anastomotic strictures after gastrectomy.

## Data Availability

The datasets generated during and/or analyzed during the current study are available from the corresponding author on reasonable request.
